# Short- or long-rest intervals during repeated-sprint training in soccer?

**DOI:** 10.1371/journal.pone.0171462

**Published:** 2017-02-15

**Authors:** F. Marcello Iaia, Matteo Fiorenza, Luca Larghi, Giampietro Alberti, Grégoire P. Millet, Olivier Girard

**Affiliations:** 1 Department of Biomedical Sciences for Health, Università degli Studi di Milano, Milan, Italy; 2 Department of Nutrition, Exercise and Sports, University of Copenhagen, Copenhagen, Denmark; 3 ISSUL, Institute of Sport Sciences, University of Lausanne, Lausanne, Switzerland; 4 Athlete Health and Performance Research Centre, Aspetar Orthopaedic and Sports Medicine Hospital, Doha, Qatar; University of Debrecen, HUNGARY

## Abstract

The present study compared the effects of two repeated-sprint training (RST) programs, differing in duration of the between-sprint rest intervals, on various soccer-related exercise performances. For 5 weeks during the competitive season, twenty-nine young trained male soccer players either replaced two of their habitual fitness conditioning sessions with RST characterized by short (5–15; n = 9) or long (5–30; n = 10) rest intervals, or served as control (n = 10). The 5–15 and 5–30 protocols consisted of 6 repetitions of 30-m (~5 s) straight-line sprints interspersed with 15 s or 30 s of passive recovery, respectively. 5–15 improved 200-m sprint time (2.0±1.5%; p<0.05) and had a *likely* positive impact on 20-m sprint performance, whereas 5–30 lowered the 20-m sprint time (2.7±1.6%; p<0.05) but was only *possibly* effective for enhancing the 200-m sprint performance. The distance covered during the Yo-Yo Intermittent Recovery Test Level 2 increased following 5–15 (11.4±5.0%; p<0.05), which was *possibly* better than the non-significant 6.5% enhancement observed in 5–30. Improvements in the total time of a repeated-sprint ability test were *possibly* greater following 5–30 (3.6±0.9%; p<0.05) compared to 5–15 (2.6±1.1%; p<0.05). Both RST interventions led to similar beneficial (p<0.05) reductions in the percentage decrement score (~30%) of the repeated-sprint ability test as well as in blood lactate concentration during submaximal exercise (17–18%). No changes occurred in the control group. In soccer players, RST over a 5-week in-season period is an efficient means to simultaneously develop different components of fitness relevant to match performance, with different benefits induced by shorter compared to longer rest intervals.

## Introduction

Repeated-sprint training (RST) is defined as “a series of short sprints (3–7 s in duration), each separated by a short recovery period (<60 s)” [1]. RST is a complex training strategy targeting the development of neuromuscular (*i*.*e*., single sprint performance) and metabolic functions (*i*.*e*., recovery between efforts), or both simultaneously. The rationale behind RST is to cause such perturbations to the muscle metabolic milieu and ion homeostasis to elicit favorable muscular (*i*.*e*., oxidative capacity, phosphocreatine recovery, H^+^ buffering) and neural (*i*.*e*., muscle activation and recruitment strategies) adaptations [2]. In a short period of time (2–5 weeks), RST offers an effective strategy to concurrently improve a range of fitness measures relevant to team sports such as explosive power, running speed, repeated-sprint ability (RSA) and high-intensity running performance [3].

Optimization of training programs requires careful manipulation of key variables, with the duration of the recovery interval or exercise-to-rest (E:R) ratio playing a central role [4] to achieve the desired changes. Regarding RSA adaptations, for instance, the effects of altering the rest interval during high-intensity exercise training have produced inconsistent results. When thirteen young male professional soccer players reduced their training volume by ~20% and replaced their habitual fitness conditioning work with 6–8 reps of 20-s all out running bouts with either 40-s (speed endurance maintenance) or 2-min (speed endurance production) rest intervals, the total time in a RSA test (15 x 40 m, 30 s of passive rest) decreased by 2.5% with long-rest training program, whereas it was *very likely* impaired following the intervention using short-rest [5]. Contrastingly, five weeks of high-repetition resistance training (15- to 20-repetition maximum, 2–5 sets) with 20-s rest periods resulted in greater improvements in RSA (5 x 6-s maximal cycle sprints) than the same training with 80-s rest periods (+12.5 *vs*. +5.4%, respectively) [6]. Additionally, while repeated-sprint performance is improved following interval training, using short-rest (1 min) does not offer any advantage over the use of long-rest (3 min) intervals when training intensity and volume are matched [7]. Referring to the RST definition [1], however, none of the aforementioned studies can be categorized as typical RST protocols. Indeed, completed efforts were either too long (*i*.*e*., 20 s [5]), of sub-maximal intensity (*i*.*e*., 92–111% of minimal running speed associated with maximal oxygen uptake (VO_2max_) [7]), or did not adopt a running/cycling mode (*i*.*e*., combination of free weights and machine [6]), or were also characterized by excessive (≥ 60 s) recovery durations [5–7].

While there is considerable variation in the RST protocols employed with regard to the E:R ratio (1:2 through 1:10) [8–11], only a limited number of studies have directly examined the effects of the duration of the recovery intervals on performance parameters with RST. In such a study, Saraslanidis et al. [12] compared the effects of two intervention programs on muscle metabolism and sprint performance: RST sessions were conducted thrice weekly over 8 weeks and comprising 2–3 sets of two 80-m sprints (~10-s efforts), differing in rest interval (10 s *vs*. 1 min). Their results suggested that training with a limited number of repeated 10-s sprints may be more effective in improving speed maintenance in 200- and 300-m runs when performed with a 1:1 rather than a 1:6 E:R ratio. However, due to the low volume of repetitions included, the involvement of fit but not well-trained participants and performance outcomes restricted to 80–300 m sprints (*i*.*e*., no RSA or endurance evaluation), the relevance/applicability of the aforementioned study to team sports may be limited.

The aim of the current investigation was therefore to compare the effects of RST, with short or long between-sprint rest intervals on a variety of soccer-related exercise performances. It has been suggested that decreasing the rest period between intervals elicits greater exercise-induced metabolic disturbances (*i*.*e*., greater activation of glycolysis [12]), thus providing a stronger stimulus for adaptations. Alternatively, it could also be argued that a severe E:R ratio (1:4 to 1:1) may impede training quality (*i*.*e*., fatigue-induced speed reduction) to an extent that could lead to smaller improvement in key performance outcomes (*i*.*e*., maximal sprinting speed) [5]. We hypothesized that RST with shorter rest intervals would augment the ability to tolerate fatigue development and to sustain supramaximal efforts, whereas RST with longer rest intervals would provide a stronger stimulus for improving the overall speed.

## Methods

### Participants

Nineteen (age 17.0 ± 1.0 yrs, height 178 ± 10 cm, body mass 69.2 ± 8.0 kg) young male sub-elite soccer players belonging to the same professional club took part in the intervention-training part of the study. All participants had a minimum of 5 yrs of experience, were regularly involved in national level competitions and none of them was under medication. Before giving their written informed consent to participate, the participants or parents/ guardians (if subject was minor) were fully informed of any possible risks and discomforts associated with the experimental procedures. The study was approved by the local ethical committee (*Commission cantonale vaudoise d'éthique de la recherche sur l'être humain*, CCER-VD 308/13) according to the code of Ethics of the World Medical Association (Declaration of Helsinki).

### Experimental design

A parallel two-group, matched-work (sprint distance), longitudinal (Pre-test, Post-test) experimental design was utilized to assess the changes in soccer-related physical performance induced by two different RST protocols. For ensuring that both groups presented equal pre-training average scores for each performance test, the players were matched according to their initial (Pre-test) athletic performances and randomly assigned to either a short- (5–15; n = 9) or a long- (5–30; n = 10) rest interval-based RST group. A control group (n = 10), performing its habitual training and equally matched for anthropometric characteristics (age 17.2 ± 0.4 yr, height 179 ± 5 cm, body mass 73.3 ± 4.9 kg) and competitive level with the two experimental groups, took part in the study. The study was conducted during the competitive season (November-January) and included: 1) Pre-training testing, 2) a 5-wk intervention training period, and 3) Post-training testing.

### Training intervention

Before the intervention, players used to perform four training sessions (*i*.*e*., Monday, Tuesday, Wednesday, Thursday) and a full-length game (Saturday) per week. The duration of all training sessions was 90–120 min and encompassed individual preparation/warm up activities, technical/tactical skills development, physical conditioning and active recovery strategies. The typical fitness work comprised injury-prevention exercises and aerobic moderate/high-intensity training on Monday, strength training and small sided-games on Tuesday, agility with changes of direction on Wednesday and quick feet drills on Thursday. During the 5-wk intervention period, the habitual interval training and agility sessions performed on Monday and Wednesday, respectively, were replaced by specific RST exercise, whereas the rest of the weekly schedule routine was kept the same as prior to the study. The detailed RST program is displayed in Table 1. Control group players continued their ordinary weekly training program as before the intervention.

**Table 1:** Repeated-sprint training program.

Week	Training session	Repeated sprint training volume	Recovery (sets)
1	1	1 set x 6 reps	-
2	2	1 set x 6 reps	-
2	3	2 sets x 6 reps	2 min
3	4	2 sets x 6 reps	2 min
3	5	2 sets x 6 reps	2 min
4	6	2 sets x 6 reps	2 min
4	7	3 sets x 6 reps	2 min
5	8	3 sets x 6 reps	2 min

The 5–15 protocol consisted of 6 repetitions of ~5-s sprints interspersed with 15 s of passive recovery, whereas 5–30 training was characterized by 6 x ~5-s sprint bouts followed by 30 s of passive recovery between repetitions. During each ~5-s bout, players carried out a single 30-m straight-line run at their maximal effort. During the first and the last training session, sprint times were recorded by the use of portable photoelectric cells (Optojump, Microgate, Bolzano, Italy). In addition, capillary blood samples were taken from the ear lobe before sprint 1 and immediately after sprint 6 of the first set of the RST protocol during both the first and the last training sessions. Blood lactate concentration ([La]) was analyzed using a portable device (Lactate Pro™, Arkray factory inc., KDK Corporation, Shiga, Japan).

All training sessions took place on artificial turf and were carefully supervised. No other physical exercise was conducted aside from the one prescribed in the soccer environment. To minimize any potential interference of external variables, the players maintained their standard lifestyle and food intake during the experimental phase.

### Performance tests

All performance tests were carried within 10 days before the commencement and after the cessation of the intervention and included: i) 20- and ii) 200-m sprint, iii) the Yo-Yo Intermittent Recovery Test Level 2 (Yo-Yo IR2), iv) a RSA test, and v) an aerobic submaximal fitness test [13].

The tests were distributed over four different experimental occasions (i.e. three during the 1^st^ week and one in the 2^nd^, 72 hrs after the game) following the same order pre- and post-intervention. Testing was always started after 15 min of standardized warm up on artificial turf (except for the 200-m sprint and the aerobic fitness test which were run on a synthetic track) under the same environmental conditions. All players were familiarized with the testing procedures (as part of their regular physical performance assessment) before commencing the study.

On the days of testing, the participants reported to the pitch or track ~3 h after having consumed a light meal. They also refrained from intense physical activity and abstained from alcohol and caffeine consumption 24 h before testing. To reduce the possible effect of diet-induced changes on muscle metabolism and subsequent exercise performance, two days before any experimental testing the participants were required to follow a nutritional strategy designed to ensure an adequate carbohydrate intake (~60% of total energy intake) and to record and replicate their individual dietary pattern during the 48-h before each testing day.

#### 20-m sprint test

Short-sprint performance was evaluated over 20-m distance. Time was recorded using photoelectric cells (Optojump, Microgate, Bolzano, Italy). Players started the sprint from a standing position with the front foot placed 10 cm behind the first timing gate. Only the best performance over three trials was considered. Each 20-m sprint was separated by 1.5 min of passive recovery.

#### 200-m sprint test

An all-out run was performed over 200 m. Time recordings were obtained as previously described for the 20-m sprint test. High reliability of the 200-m sprint test was reported (coefficient of variation: 0.8 ± 0.7%) in players of similar standard [5].

#### Yo-Yo IR2 test

The Yo-Yo Intermittent Recovery Test Level 2 consisted of 2 x 20-m shuttle runs at increasing speeds, interspersed with 10 s of active recovery, controlled by audio signals. The test was terminated when the participant was no longer able to maintain the required speed and the distance achieved was recorded as result [14].

#### RSA test

The RSA test consisted of 15 repetitions of 40-m straight-line sprints interspersed with 30 s of passive recovery [15, 16]. Time was measured with photoelectric cells (Optojump, Microgate, Bolzano, Italy). Total sprint time (RSA_t_) for all 15 sprints was determined. In addition, in order to quantify fatigue during the RSA test, the percentage decrement score (RSA_Sdec_) was calculated as follows [17]:

$$RSA_{Sdec}=\left[\frac{\left(S_{1}+S_{2}+S_{3}+\ldots +S_{final}\right)}{S_{best}\times number\;of\;sprints}-1\right]\times 100$$

The high reliability reported for the current RSA test (coefficients of variation for RSA_t_ and RSA_Sdec_ of 1.2 ± 0.9% and 16.8 ± 14.9%, respectively [5]) in players of similar standard is also in line with reliability scores of other RSA protocols [10, 18].

#### Aerobic fitness test

The aerobic fitness test consisted of 6 min running at a constant speed of 13.5 km/h [13]. A capillary blood sample was collected from the ear lobe immediately after the end of the test and [La] was analyzed (see *Training intervention*).

### Statistical analyses

The normality distribution of each variable was examined with the Shapiro-Wilk test. An unpaired t-test was used to ensure that no between-group differences existed in the Pre-test measurements. Data were analyzed using a two-factor repeated-measure ANOVA with one within factor (time: Pre-test *vs*. Post-test) and one between factor (group: 5–15 *vs*. 5–30). When a significant main effect was detected, a Student-Newman-Keuls post hoc analysis was applied for pairwise multiple comparison. A paired t-test was applied to evaluate Pre-to-Post differences in single sprint performances of the RSA test.

In addition to the null-hypothesis test, to allow a better interpretation of the results, the practical significances were verified using a statistical approach based on the magnitudes of change [19]. For between- and within-group comparisons, the chances that the true mean changes following each training program were *beneficial* (*i*.*e*., greater than the smallest worthwhile change, SWC [0.2 multiplied by the between-subject standard deviation]), *unclear/trivial* or *harmful* for performance were calculated. Quantitative chances of *beneficial*, *trivial*, or *harmful* changes were evaluated qualitatively as follows: 25–75%, possibly; 75–95%, likely; 95–99%, very likely; and >99%, almost certainly. If the chances of having *beneficial* or *harmful* performance changes were both >5%, the true difference was defined as *unclear* [19]. Moreover, the effect size (ES) of changes in each performance parameter was calculated using the pooled Pre-training standard deviation [20] and was utilized to express within-group standardized changes and between-group standardized differences. The level of statistical significance was set for all analyses at p < 0.05. Raw data are presented as means ± SD, whereas relative changes are means ± 90% confidence intervals.

## Results

### Training compliancy and training responses

The nineteen players recruited for the intervention had a training compliancy >85%, and therefore were all included in the final analysis. No injuries occurred during the intervention period. No differences (p > 0.5) were observed between the two RST groups in any of the pre-test measurements.

During the first 5–15 training session, the average running speed from sprint 1 to 6 decreased by 2.7% (p = 0.048), while it only tended to be reduced during the last training session. On this occasion, the running speed reached during the final repetition (6^th^ sprint) was faster (p = 0.049) during the last (23.4 ± 0.5 km/h) compared to the first (22.7 ± 0.5 km/h) training session. In 5–30, no significant changes in speed were observed between sprint 1 and 6 during both the first and the last training session (Table 2).

**Table 2:** Average sprint running speed and blood lactate response to one bout of either 5–15 or 5–30 repeated-sprint training protocol during the first and the last training session.

		5–15		5–30	
		First training session	Last training session	First training session	Last training session
Average running speed (km/h)	Sprint 1	23.3 ± 0.8	24.0 ± 0.8	23.7 ± 0.8	24.0 ± 0.9
	Sprint 6	22.7 ± 0.5*	23.4 ± 0.5^#^	23.4 ± 0.8	24.0 ± 1.3
[La] (mmol/L)	Before sprint 1	3.1 ± 0.8	3.6 ± 1.1	3.5 ± 1.1	3.4 ± 1.0
	After sprint 6	9.3 ± 1.6^§^	9.3 ± 2.0^§^	6.6 ± 1.8^†^^§^	6.0 ± 1.3^†^^§^

^#^ Different (p < 0.05) from the First training session.

^†^ Different (p < 0.05) from 5–15.

* Different (p < 0.05) from Sprint 1.

^§^ Different (p < 0.05) from Before sprint 1.

In 5–15, during the first training session, [La] increased (p < 0.05) from 3.1 ± 0.8 mmol/L before sprint 1 to 9.3 ± 1.6 mmol/L following sprint 6, which were the same after the intervention. In 5–30, post-exercise [La] increased (p < 0.001) about twofold but it was significantly lower compared with 5–15 during both the first (p<0.05) and the last training session (p<0.01) (Table 2).

### Performance

**Fig 1:**
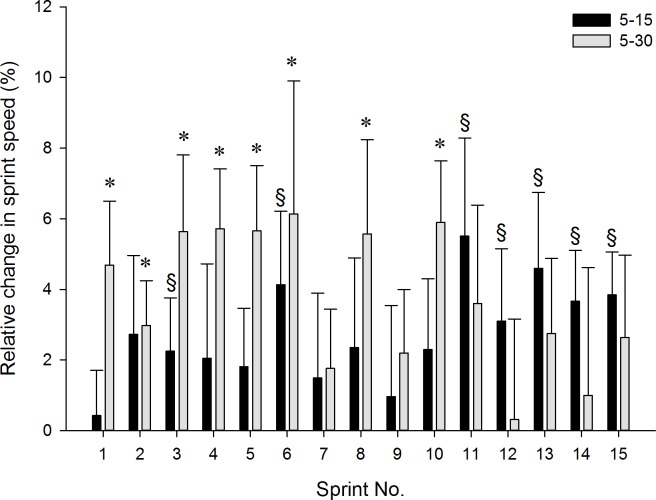
Relative changes (% ± 90% CI) in average running speed during the sprints of the RSA test. ^§^Significant Pre-to-Post difference in 5–15 (p < 0.05). *Significant Pre-to-Post difference in 5–30 (p < 0.05).

Performance scores for the RST groups are reported in Table 3. 5–30 improved the 20-m sprint time by 2.7% (p = 0.004), whereas only a tendency was observed in 5–15 (1.5%; p = 0.086). In 5–15, 200-m sprint and Yo-Yo IR2 test performance improved by 2.0% (p = 0.02) and 11.4% (p = 0.005), respectively, while no changes occurred in 5–30 (p>0.097). Both 5–15 and 5–30 induced improvements in RSA_t_ (p<0.001) and RSA_Sdec_ (p<0.05) following the intervention. In addition, 5–15 improved performance in the 3^rd^, 6^th^ and from the 11^th^ to 15^th^ sprint of the RSA test (p<0.05), whereas speed increments were noted from the 1^st^ to the 6^th^ and in the 8^th^ and 10^th^ sprint in 5–30 (p<0.05) (Fig 1). After the aerobic fitness test, [La] was 0.9 ± 0.8 and 1.1 ± 1.4 mmol/L lower (p<0.05) following 5–15 and 5–30 interventions, respectively, with no difference between groups.

**Table 3:** Changes in performance following the 5–15 and 5–30 repeated sprint training protocols.

	5–15 (n = 9)						5–30 (n = 10)					
	Pre	Post	% change ±90% CI	Standardized change (Cohen’s d ±90% CI)	% chances of detrimental/trivial/beneficial effect	Qualitative inference	Pre	Post	% change ±90% CI	Standardized change (Cohen’s d ±90% CI)	% chances of detrimental/trivial/beneficial effect	Qualitative inference
20-m (s)	3.30 ± 0.09	3.25 ± 0.06	-1.5 ± 1.3	-0.51 ± 0.45	1/10/89	Likely	3.29 ± 0.08	3.21 ± 0.08*	-2.7 ± 1.6	-1.12 ± 0.68	0/2/98	Very likely
200-m (s)	29.60 ± 0.90	29.01 ± 0.83*	-2.0 ± 1.5	-0.60 ± 0.44	1/6/93	Likely	29.56 ± 1.11	29.17 ± 1.12	-1.3 ± 1.4	-0.32 ± 0.34	1/25/74	Possibly
Yo-Yo IR2 (m)	1000 ± 169	1111 ± 171*	11.4 ± 5.0	0.56 ± 0.24	0/1/99	Very likely	1016 ± 217	1072 ± 156	6.5 ± 7.3	0.29 ± 0.32	1/30/69	Possibly
RSA_t_ (s)	92.91 ± 4.66	90.47 ± 4.24*	-2.6 ± 1.1	-0.47 ± 0.21	0/2/98	Very likely	91.45 ± 4.35	88.22 ± 4.65*	-3.6 ± 0.9	-0.70 ± 0.17	0/0/100	Almost certainly
RSA_Sdec_ (%)	5.90 ± 2.21	4.12 ± 1.57*	-30.6 ± 15.1	-0.72 ± 0.43	0/3/97	Very likely	5.19 ± 2.09	3.67 ± 1.67*	-30.4 ± 13.2	-0.86 ± 0.45	0/1/99	Very likely
Aerobic fitness test (mmol/L)	5.49 ± 2.22	4.35 ± 1.31*	-17.5 ± 7.2	-0.64 ± 0.29	0/1/99	Very likely	5.71 ± 1.53	4.77 ± 1.59*	-18.0 ± 11.2	-0.40 ± 0.27	0/10/90	Likely

* Significant difference from Pre (p < 0.05)

None of the performance variables differed (p>0.05) between pre-test and post-test in the control group (20-m: 3.11 ± 0.09 *vs*. 3.05 ± 0.33 s; 200-m: 26.83 ± 0.84 *vs*. 28.51 ± 0.92 s; Yo-Yo IR2: 688 ± 146 *vs*. 704 ± 181 m; RSA_t_: 89.19 ± 1.48 *vs*. 89.21 ± 1.53 s; RSA_Sdec_: 5.55 ± 2.81 *vs*. 5.52 ± 2.59%; aerobic fitness test: 5.12 ± 1.74 *vs*. 4.98 ± 1.92 mmol/L).

### Magnitude-based approach

Relative and standardized changes and qualitative inferences from within-group analysis are presented in Table 3.

**Fig 2:**
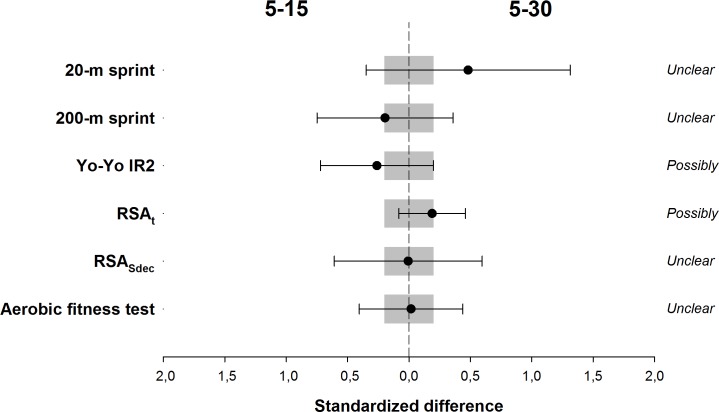
Effects of 5–15 compared with 5–30 training on 20- and 200-m sprint time, Yo-Yo IR2 test performance, total sprint time (RSA_t_) and percentage decrement score (RSA_Sdec_) of the repeated-sprint ability test, and aerobic fitness test performance. Data are presented as standardized difference (Cohen’s d) ± 90% CI.

Between-group changes are presented in Fig 2. Changes in Yo-Yo IR2 test performance were *possibily better* following 5–15 compared to 5–30. Conversely, 5–30 induced *possibly greater* improvements in RSA_t_ than those observed with the 5–15 protocol. Differences in the changes in 20-m, 200-m, RSA_Sdec_ and aerobic fitness test were *unclear*.

## Discussion

The major findings of the present study were that 5–15 improved the 200-m sprint and Yo-Yo IR2 performance whereas 5–30 reduced the time in a 20-m sprint test. Both RST programs evoked positive changes in aerobic fitness and RSA (RSA_t_ and RSA_dec_) with *possibly* greater improvements in RSA_t_ following 5–30 compared to 5–15.

### Training responses

Short rest intervals (*i*.*e*., 15 s) between maximal efforts (30 m or ~5 s) during training resulted in an inability to maintain sprint performance constant (RSA was not altered in 5–30), with this effect becoming less evident during the last training session. Shorter recoveries also placed greater emphasis on glycolytic energy production, as demonstrated by significantly greater elevation in [La] values in 5–15 *vs*. 5–30, yet with unchanged [La] values following both training interventions. Of importance, our RST intervention was implemented ‘in-season’ and utilized a design whereby RST replaced the usual field-based conditioning work to keep training load similar before and during the intervention period as well as between groups in a cohort of trained youth soccer players.

### 20-m sprint

A *possibly moderate* effect of RST on 20-m sprint performance (mean difference; ±95% confidence limits: -0.07 s; ±0.08) has been highlighted in a recent meta-analysis [3]. In our study, long-rest (*i*.*e*., 30 s) intervals between sprints allowed for the maintenance of a higher sprinting speed across repetitions (during training) and resulted in a significant improvement in 20-m sprint performance, which on the contrary did not significantly change when employing short-rest (*i*.*e*., 15 s) periods. This is consistent with Fernandez-Fernandez et al. [9] who showed that 6 weeks of RST (18 training sessions in total), employing 3 sets of ten ~5-s shuttle sprints interspersed with relatively short (20 s) recoveries between repetitions (1:4 E:R ratio), had no effect on 20-m sprint times. Taken together these findings seem to suggest that the magnitude of change is a direct consequence of the RST protocol or E:R ratio utilized, with the reiteration of maximal speed during each exercise bout constituting a key element for enhancing single sprint performance. The mechanisms underpinning such improvement could also be, among others, ascribed to beneficial changes in the patterns of muscle activation (*i*.*e*., reduced co-contractions, larger recruitment of the gluteal muscle group contributing to a more efficient hip extension to achieve faster speed) [21].

### 200-m sprint

After the intervention, a significant improvement in 200-m sprint performance was detected in 5–15, while only *possible* beneficial changes were noted in 5–30. Consistently, greater effect in enhancing 200-m sprint time was observed after speed endurance maintenance (1:3 E:R ratio) training compared with speed endurance production (1:6 E:R ratio) training [5] or when 10-s repeated sprints were carried out with 10-s (1:1 E:R ratio) rather than 60-s (1:6 E:R ratio) rest intervals [12]. Overall, this indicates that short- *vs*. long-rest training program provides stronger stimuli for improving the ability of active muscles to preserve speed decrement and sustain short-duration supra-maximal exercise averaging 30 s, such as during the 200-m run when glycolysis is the predominant energy source [22]. The mechanisms underlying the greater improvement in 200-m performance with narrower E:R ratios during training might therefore include a greater activation of glycolysis, caused in part by the limited resynthesis of phosphocreatine due to shortened recovery. In support of this, the drop in phosphocreatine and the increases in products of glycogen breakdown (glucose-6-phosphate and fructose-6-phosphate) after two 80-m sprints following 8 weeks of sprint training were greater in the short-rest *vs*. long-rest training program [12].

### High-intensity intermittent exercise capacity

The Yo-Yo IR2 test performance, explaining 40% of the variance in high-intensity game distance in soccer [23], not only highly taxes the aerobic but also the anaerobic energy system during accelerations inherent with consecutive directional changes [14]. In the present study, the distance covered during the Yo-Yo IR2 test increased significantly by 11.4% from pre- to post-training in 5–15, which represented a *possible better* change than the non-significant 6.5% increase observed in 5–30. A plausible explanation for the larger performance improvement with short- *vs*. long-rest training program could relate to a stronger stimulation of both the aerobic and anaerobic (lactic) metabolism [1]. Irrespective of the duration of the rest intervals, training-induced gains in high-intensity intermittent performance are consistent with those of previous studies involving fit individuals (+8–10% [24, 25]) but lower than improvements ranging from 14 to 29% generally seen after 2–7 weeks of RST in team- sport players (Yo-Yo IR2 in soccer [10, 26]; rugby [27]; field hockey [28]) and racket- (Hit and Turn tennis test in tennis [9]). In addition to differences in RST content/modality (*i*.*e*., E:R ratio, straight line/shuttle runs), discrepant training responses between our results and literature findings may partially be related to the season period (competitive season *vs*. pre-season [29]) and the characteristics (sub-elite vs. amateur players or under 18 *vs*. other age groups [30]) of the tested individuals.

### Repeated-sprint ability

Despite the different exercise to rest ratio, the current RST programs induced substantial improvements in performance fatigability (*i*.*e*., similar reductions in RSA_Sdec_ averaging ~30%) across the fifteen running sprints. Such enhanced ability to recover between consecutive sprints may relate to the specificity of RST for inducing positive changes in ion homeostasis and buffering capacity and promoting an oxidative phenotype in skeletal muscle [2]. Both training programs also conferred significant beneficial changes in total sprint time (RSA_t_), with the improvements being *possibly greater* in 5–30 compared to 5–15. Overall, RSA gains observed here were in the higher range for RSA_Sdec_ (0–39%) and in the lower range for RSA_t_ (1.5–8.8%) compared to what have been previously reported in team- [8, 10, 11, 27, 31] and racket-sport players [9] as well as in recreationally trained individuals [24, 25, 32, 33] after similar RST regimens conducted over 4–7 weeks. In a meta-analysis, a *possibly moderate* beneficial effect of RST on RSA (effect size -0.62; 95% confidence limits ± 0.25) was found from the analysis of 8 studies [3]. Neuromuscular adaptations or enhancements of maximal aerobic power (*i*.*e*., increased oxygen delivery to muscle tissue, concentration of aerobic enzymes, mitochondrial size and number, and capillary density) or anaerobic capacity (*i*.*e*., enhanced phosphocreatine resynthesis, upregulation of key glycolytic enzymes, increased lactate clearance and inorganic phosphate removal as well as increased muscle buffer capacity) have been identified as crucial adaptive factors for improved tolerance to repeated maximal efforts [2, 34]. Importantly, improvement in RSA_t_ was *possibly* greater in 5–30 compared to 5–15. This observation suggests that implementing longer rest intervals during RST may more largely up-regulate some of the aforementioned mechanisms, and thereby, induce superior benefits in repeated-sprint performance. Similar improvement in performance fatigability (RSA_Sdec_) following both training interventions occurred concurrently with larger gains seen in 5–30 for both single sprint (first effort of the repeated-sprint series) and mean performance (RSA_t_). This novel information indicates that RST with a long-rest rather than short-rest may be more effective at improving RSA in youth soccer players.

Another interesting observation is that the relative change in sprint speed was greater during the first third of the RSA test in 5–30, whereas greater (speed) improvements were noted in the last third of the repeated sprint series in 5–15 (Fig 1). Although the contribution of oxidative phosphorylation to total energy expenditure during a single short sprint is limited, the level of aerobic ATP provision progressively increases as sprints are repeated [35] such that aerobic metabolism may contribute as much as 40% of the total energy supply during the final repetitions of a repeated-sprint series [36]. With VO_2max_ being reached during the latter sprints of an RSA test resembling the one performed here (15 x 40 m, 25-s rest [16]), this suggests that VO_2max_ may limit the contribution of aerobic metabolism. Also, larger RST-induced gains for 5–15 *vs*. 5–30 in the distance covered during the Yo-Yo IR2 provide indirect evidence that increasing VO_2max_ to a greater extent may allow for a higher aerobic contribution during the latter sprints, potentially minimizing fatigue (*i*.*e*., greater relative change in sprint speed).

### Aerobic capacity

A lower [La] response was observed post-training after running at constant speed. This can be the result of both a greater clearance of lactate from the blood and lower production of lactate (no possible distinction here), which has been associated with an increase in the activity of oxidative enzymes and an elevated fat oxidation [37]. Interestingly, an increase in the content of monocarboxylate transporters 1 (no change in isoform 4), a dominant regulator of muscle pH during and after repeated intense exercise, has been observed in response to a 8-wk period of intermittent-sprint training (30 sessions including 15 x 6-s sprint with 1-min active recovery) [24]. Whatever the exact underpinning mechanisms of pH regulatory systems are, our results showed that RST can reduce [La] at a given submaximal fixed (13.5 km/h) intensity. However, altering the rest interval during training (5–15 *vs*. 5–30) did not affect the magnitude of changes in [La]. That said, improved exercise testing prescription might require that anaerobic speed reserve (*i*.*e*., the difference between maximal sprinting speed and minimal running speed associated with VO_2max_ [1]) is systematically re-assessed during pre- and post-test sessions for the purpose of setting individualized running speeds.

## Conclusion

To our knowledge, this study is the first to compare the effect of two RST programs, differing in rest intervals between sprints (1:3 *vs*. 1:6 E:R ratios), on various types of soccer-related exercise performances. Our main findings are as follows: first, whilst 5–15 improved 200-m and had a *likely* positive impact on 20-m sprint performance, 5–30 lowered the 20-m sprint time but was only *possibly* effective for enhancing the 200-m performance. Second, the distance covered in the Yo-Yo IR2 test increased after 5–15 which was *possibly* better than the non-significant change observed in 5–30. Third, both RST interventions led to similar significant beneficial reductions in [La] after submaximal exercise as well as in the percentage decrement score and total time in a RSA test, with *possibly* greater improvements in RSA_t_ following 5–30 compared to 5–15. In youth professional soccer players, RST over a 5-wk in-season period is an efficient means to simultaneously develop different components of fitness relevant to match performance, with different benefits induced by shorter or longer rest intervals.

### Practical applications

Despite the proven efficiency of RST for improving a wide range of fitness components of soccer performance there is still considerable debate on the most appropriate training practice to adopt (*e*.*g*., for instance to maximize RSA gains [2, 38]). The observation that short- *vs*. long-rest intervals induce specific adaptations, however, supports the common view that there is not one type of training that can be recommended to better improve physical performance in team sports [2, 5]. Additionally, direct comparisons between the effectiveness of RST and high-intensity interval training have shown that the latter may be a more effective intervention to improve RSA mean time in young handball players [32] as well as sport-specific aerobic fitness in tennis players [9] and healthy individuals [24], but opposite results have also been reported [10]. Furthermore, in rugby players, combined RST and resistance (squat) training with superimposed vibration was suggested to be more advantageous for improving repeated-sprint performance than RST alone [31]. Finally, by adding hypoxic stress to RST, larger short-term (2–6 weeks) repeated-sprint performance enhancements have been reported compared to similar training at sea level [26, 27, 39]. While the aforementioned considerations would suggest that a combination of different training practices/methods is probably the most effective approach, it remains to be determined how to best manipulate RST variables with also efficient periodization of conditioning sessions in the busy schedule of players to reach the desired training adaptations.

### Perspectives

Future RST studies with a wider spectrum of E:R ratios and including muscle biopsy samples are needed to determine what is the optimal trade-off between maintenance of speed across sprint series (*i*.*e*., opportune fatigue level) and large intramuscular metabolic and/or acid-base disturbances during exercise (*i*.*e*., meaningful training stimulus). If a different approach is needed and a sensitive reduction of exercising volume is expected (*i*.*e*., congested or tapering periods), training with shuttle sprinting, allowing to reach a level of fatigue similar to that obtained in straight sprinting, can be considered as a valuable alternative but would require careful management of neuromuscular load [26, 40]. Even more crucial for match physical performance is the ability of players to accelerate while changing direction and orientation [41] and thereby the need to design effective training methods (*i*.*e*., individual drills or small-sided games with the use of appropriate E:R ratio [42]) specifically aimed at improving these repeated sprint abilities.
